# Immunology IV: Clinical Applications in Health and Disease by Joseph A. Bellanti

**DOI:** 10.1097/WOX.0b013e3182641db0

**Published:** 2012-08-15

**Authors:** Sergio Bonini

**Affiliations:** 1 Second University of Naples, Naples, Italy

## 

1063 pages, 800 color figures, Hardcover, I Care Press, Bethesda MD, 2012

Price: 99 US$, including a 2-year subscription to the online version

For orders: http://www.immunologycenter.org

The final comment I usually make when reviewing a book is about its cost. On this occasion, however, I should like to start just with this because readers will finally welcome a book with a low price but, mainly, with an extraordinary cost to value ratio.

In fact, why should we spend a fortune to buy expensive books, which rapidly become out-dated, when Internet access offers us a massive and updated scientific information? The answer is often confined to the pleasure of senses that only an elegant, paper-smelling concrete object with attractive colored pictures may give. This is certainly the case for this hardcover textbook, 1063 pages of high-quality paper and 800 color illustrations.

But this is not the only reason that fired on my enthusiasm for the book of Joseph A. Bellanti.

First, the editor and the 2 coeditors (Aleandro Escobar-Gutierrez and George E. Tsokos), certainly aware of the potential limitations of the printed scientific information versus the electronic one, offer in addition to this completely rewritten fourth 2012 Edition of one of the most famous allergy and clinical immunology textbooks, a complementary online version included in the cost of the book. This allows interactive multilevel learning and teaching by providing animations, hyperlinks, clinical case studies with questions and answers and, mainly, access to a continuously updated version of the book.

Second, the authors of the 25 chapters were selected among the most qualified experts for each topic, without anyway compromising the homogeneity of the textbook, made possible by the direct participation of the editor among authors of several chapters. This avoids the omissions or repetitions often found in many textbooks.

Finally, the book fully respects the transversal nature of the discipline of allergy and clinical immunology by covering, from bench to bedside: cells, cytokines and mediators of the immune response, immunodeficiencies, allergic diseases, autoimmune diseases, immune response to cancer, lymphoproliferative diseases, and transplantation immunology. The above chapters are complemented by specific chapters on laboratory tests and on the most recent treatments, including vaccines, each one enriched by excellent summarizing tables (for instance, I never saw such a useful comprehensive table as the 5-page table on immune response modifiers).

I have to disclose a potential conflict of interest in reviewing this book: I am certainly influenced by the great admiration I have for Joseph Bellanti, as a scientist, as a doctor, and as one of the pioneer mentors of the discipline of allergy and clinical immunology, from whom I personally learned the first basics at the beginning of my career. However, I do not feel it influenced in anyway my very positive opinion on what Bellanti and coworkers were able to achieve.

In fact, I do think that the textbook of Joseph A. Bellanti is the ideal reference not only for pregraduate and postgraduate students and residents but also a crucial resource for all researchers and doctors

Sergio Bonini

Second University of Naples, Naples, Italy

